# Extent of cortical involvement in amyotrophic lateral sclerosis – an analysis based on cortical thickness

**DOI:** 10.1186/1471-2377-13-148

**Published:** 2013-10-18

**Authors:** Johannes Thorns, Henk Jansma, Thomas Peschel, Julian Grosskreutz, Bahram Mohammadi, Reinhard Dengler, Thomas F Münte

**Affiliations:** 1 Department of Neurology, University of Lübeck, Ratzeburger Allee 160, Lübeck, 23538, Germany; 2 Department of Psychiatry, Medical School Hannover, Hannover, Germany; 3 Department of Neurology, University of Jena, Jena, Germany; 4 Department of Neurology and Clinical Neurophysiology, Medical School of Hannover, Hannover, Germany

**Keywords:** ALS, Cortical thickness, MRI

## Abstract

**Background:**

Besides the defining involvement of upper and lower motor neurons, the involvement of extramotor structures has been increasingly acknowledged in amyotrophic lateral sclerosis (ALS).

**Methods:**

Here we investigated a group of 14 mildly to moderately affected ALS patients and 14 age-matched healthy control participants using cortical thickness analysis. Cortical thickness was determined from high resolution 3D T1 magnetic resonance images and involved semiautomatic segmentation in grey and white matter, cortical alignment and determination of thickness using the Laplace method. In addition to a whole-cortex analysis a region of interest approach was applied.

**Results:**

ALS patients showed regions of significant cortical thinning in the pre- and postcentral gyri bilaterally. Further regions of cortical thinning included superior and inferior parietal lobule, angular and supramarginal gyrus, insula, superior frontal, temporal and occipital regions, thus further substantiating extramotor involvement in ALS. A relationship between cortical thickness of the right superior frontal cortex and clinical severity (assessed by the ALS functional rating scale) was also demonstrated.

**Conclusions:**

Cortical thickness is reduced in ALS not only in motor areas but in widespread non-motor cortical areas. Cortical thickness is related to clinical severity.

## Background

Amyotrophic lateral sclerosis (ALS) is a neurodegenerative disease with upper and lower motor neuron involvement as its clinical hallmarks. Dating back to very early observations that a significant percentage of ALS patients develop a dementia it has been clear that extramotor involvement can occur. That such involvement is the rule rather than the exception has been underscored by findings from neuropsychological [1-4] and neuroimaging [5-9] investigations. The recent discovery that the C9ORF72 hexanucleotide repeat can cause both, frontotemporal dementia and ALS as well as overlapping syndromes, further underscores that the molecular underpinnings of ALS might damage extramotor and motor parts of the nervous system [10]. The question therefore arises, to what extent disturbances of non-motor functions are reflected in changes of cortical structure. Primary motor and extramotor involvement has been demonstrated repeatedly by voxel based morphometry (VBM) [11-13], although other studies did not report atrophy [14,15].

VBM is a fully automated, operator-independent technique that allows the voxel-wise comparison of segmented grey and white matter images between two groups of subjects [16-19]. As such, it has the advantages of being fast and independent of any observer rating or a priori hypothesis on the location of the structural abnormality. There are several limitations of this approach, however [18,20]. For example, VBM simultaneously reflects size, position and morphology. As nonlinear spatial normalization and spatial filtering is used to accommodate anatomical variations between brains, there is the danger of missing important differences, in particular when these are circumscribed. Moreover, VBM reveals differences in grey matter density, which may not equate in a simple way to atrophy.

In the present investigation we therefore employed an alternative to VBM, cortical thickness analysis (CTA). CTA is based on a semiautomatic/interactive segmentation of brain white and grey matter followed by a determination of the thickness of the cortical layer [21-24]. Thickness values are then entered into standard between group statistics allowing a comparison for the entire cortical surface. Neuropathological studies have described atrophy involving the precentral gyrus with loss of Betz cells and gliosis in ALS [25,26] which lead us to suspect a decreased cortical thickness in ALS. Also, cortical thinning of the motor cortex has been described recently in ALS [27-30] and primary lateral sclerosis [31]. Verstraete et al. [28,29] could demonstrate that cortical thinning in temporal regions was related to faster clinical progression, whereas in a recent study by Agosta et al. [30] age and cortical thinning in sensorimotor cortex was related in ALS patients but not in controls. Interestingly, no relationship was found between clinical severity and cortical thickness. Finally, in a recent study by Schuster et al. [32] clinical variables were related to cortical thinning of the primary motor cortex (PMC). Upper motor neuron (UMN) signs in the bulbar region were associated with bilateral thinning within the bulbar segment on the motor cortex, whereas UMN signs of the extremities were associated with thinning in the limb segment of the motor cortex.

In the present investigation, we asked specifically whether other areas in addition to the motor cortex are affected with regard to cortical thinning. We were further interested in whether CTA is more sensitive to cortical changes in ALS than VBM and performed CTA on a sample of ALS patients and controls that has previously been reported in a VBM study [13].

## Methods

### Patients

The study was approved by the ethics committee of Hannover Medical School. All participants gave their written informed consent prior to their inclusion in the study.

The original patient group comprised 17 subjects (3 women) classified as having definite, probable or possible ALS according to the revised El Escorial criteria [33] at the time of testing. All but one patient had progressed to definite or probable ALS on follow up. Data quality of the scans did not allow reliable differentiation between grey and white matter in three patients. For the remaining 14 patients mean (± S.D.) duration since onset of symptoms, henceforth disease duration, was 23.1 ± 7.7 months, mean age was 58.4 ± 13.1 (range 31–77) years and duration of education in years was 12.8 ± 2.6. Seventeen age matched healthy participants were recruited as a control group. Of these, only those 14 participants that most closely matched the 14 remaining patients with regard to age (57.9 ± 11.8, p = 0.86), sex (patients: 2 women, controls: 3 women), and education (controls 11.9 ± 3.1, p = 0.79) were further analyzed. Neither patients nor controls had a history of cerebrovascular disease, longstanding hypertension or inflammatory disease of the central nervous system. All patients received riluzole treatment but no psychoactive drugs. Their score on the revised ALS functional rating scale [34,35] (ALSFRS-R) was 40.6 ± 4.7 points.

### Clinical data

A number of clinical data were obtained to correlate these with cortical thickness. ALSFRS-R progression rate per month was calculated for the total disease duration [PR/mth(t)] and the six month period prior to MRI [PR/mth(6)]. Sum scores of Medical Research Council (MRC) muscle strength were taken for the upper extremity (shoulder abduction, inward and outward rotation; elbow flexion and extension; lower arm pronation and supination; wrist flexion and extension; finger flexion, extension, abduction and adduction; 5th finger abduction and thumb opposition; highest possible score 75 points) and the lower extremity (hip strength; knee flexion and extension; foot flexion, extension, inversion and eversion; toe flexion and extension; best possible score 45 points). Bulbar involvement was described as a sum score of dysarthria (0 = no, 1 = yes), dysphagia (0 = no, 1 = yes), eyelid closure, mouth closure, tongue movement and palate elevation (0 = normal, 1 = reduced, 2 = weak, 3 = absent) with a lowest possible score of 14. Reflexes were summed by counting the number of muscles scoring 3 or 4 on the NINDS reflex scale for the biceps, triceps, brachioradialis, finger flexor, quadriceps and gastrocnemius muscles on both sides and masseter muscle (maximum score 13). Spasticity was described as none (0), noticeable (1), pronounced (2), barely to overcome (3) and not to overcome (4) with a worst score of 32 (arm, hand, upper thigh, lower thigh both sides). A positive Babinski sign was counted separately for each side (worst score of 2). The clinical characteristics of the patients are summarized in Table 1.

**Table 1:** Clinical data

**Pat.**	**Age**	**Sex**	**Dur.**	**RUE (75)**	**LUE (75)**	**RLE (45)**	**LLE (45)**	**Bulbar (0–14)**	**Babinski (0–2)**	**Spasticity (0–32)**	**Reflexes (0–13)**	**ALSFRS-R**	**ALSFRS-R PR/M6**	**ALSFRS-R PR/M(t)**
1	59	m	30	68	69	41	41	2	0	0	0	43	0.5	0.2
2	59	m	13	75	64	31	27	0	0	0	0	37	1.8	0.8
3	31	m	17	64	62	45	45	0	2	0	12	42	1.2	0.4
4	68	m	22	75	73	45	42	2	2	4	4	40	0.7	0.4
5	65	m	25	72	75	26	16	0	1	0	6	37	0.7	0.4
6	53	m	38	74	75	26	23	0	0	0	0	43	0.2	0.1
7	34	w	18	70	71	45	45	4	2	0	12	39	1.0	0.5
8	62	m	18	63	73	45	45	4	0	0	12	40	0.7	0.4
9	49	m	19	54	55	45	45	0	2	0	0	41	0.3	0.4
10	60	m	30	58	57	45	45	2	2	0	5	33	1.5	0.5
11	77	m	34	70	71	22	37	0	2	2	12	33	1.7	0.4
12	63	w	12	75	75	45	45	3	0	0	11	45	0.2	0.3
13	66	m	23	61	71	44	44	0	1	0	0	48	0.0	0.0
14	72	m	25	69	71	40	41	3	2	4	6	48	1.0	0.3

Pat. = patient; Dur. = duration in months; m = man, w = woman; RUE, LUE, RLE, LLE: right upper, left upper, right lower, left lower extremity; ALSFRS-R: ALS functional rating scale- revised, PR/M6 progression rate for the 6 months prior to MR-scanning, PR/M(t) progression rate per month for the entire duration of the disease.

### Image acquisition

Images were acquired on a neuro-optimized 1.5-T GE Signa Horizon LX (General Electric Company, Milwaukee, WI, USA) using a 3-dimensional T1-weighted spoiled gradient recalled echo (SPGR) sequence generating 124 contiguous sagittal slices (RT 24 ms; TE 8 ms; flip angle 30°, 2 averages, acquisition time 13′10′, in plane resolution 0.97 × 0.97 × 1.5 mm^3^). During scanning, all participants were comfortably placed and their heads were fixated within the headcoil with special cushions. All subjects received additional T2-weighted images to exclude any ischemic or inflammatory lesions. These were normal in all participants.

### Preprocessing and advanced segmentation analysis

The entire analysis was performed using Brainvoyager QX 1.8-1.10 (Brain Innovation, Maastricht, The Netherlands). Data was resampled to achieve 1 × 1 × 1 mm resolution, aligned to the AC-PC line and transformed into Talairach standard space. Prior to cortical thickness analysis, data had to be resampled once more to 0.5 mm iso-voxel using sinc interpolation. The brain was then segmented from surrounding head tissue using an automatic “brain peeling” tool [36] and subcortical structures and the cerebellum were removed using a mask. Tissue contrast and homogeneity were enhanced by converting the grey-scale from 8 bit to 16 bit resolution and by using a sigma filter. The border between white and grey matter was segmented automatically using an adaptive region growing step using locally computed histograms and gradient information. Subsequently, the border between grey matter and cerebrospinal fluid border was segmented using a dilation process beginning at the white matter–grey matter boundary and moving towards the CSF boundary. This process was controlled by computed gradient fields and histogram analysis of grey matter–CSF threshold values. The aforementioned segmentation steps proved to be unreliable in several regions of the brain, leading, for example, to bridges across sulci. Thus, extensive interactive correction was carried out by author JT who was blinded with regard to whether or not a given brain belonged to a patient or a control participant.

### Cortical alignment

To further improve the spatial correspondence between participants’ brains, reconstructed cortices were aligned using curvature information reflecting the gyral/sulcal folding pattern. This approach has been shown to substantially reduce anatomical variability [37]. The reconstructed folded cortical representations of each participant were morphed into a spherical representation (separately for each hemisphere) providing a parameterizable surface suitable for across-subject non-rigid alignment. Each vertex on the sphere corresponded to a vertex of the folded cortex. The curvature information was preserved as a curvature map on the spherical representation and was smoothed along the surface to provide spatially extended gradient information for intercortex alignment. The mean squared differences between the curvature of a source and a target sphere were minimized during the iterative alignment process [36,38].

Cortical thickness maps for each subject were calculated and measurements were performed using the Laplace method [23] as implemented in Brain Voyager Qx. For the whole-cortex analysis the correction for multiple comparisons was performed using cluster-size thresholding. The procedure started with setting a threshold (manually) for the map relative to the contrast of interest with a p < 0.05 (uncorrected). Next, the intrinsic smoothness of the resulting map was evaluated automatically [36,39]. The final result was a minimum cluster size threshold (1 mm^2^) for the current map to achieve a corrected p value of < 0.045.

### Region-of-interest analysis of cortical thickness maps

While group differences in cortical thickness measurements can be mapped at the vertex level, the statistical power of this approach is limited because of the multiple comparisons problem. We therefore also explored cortical thickness in certain regions (or more precisely cortical patches) of interest. We used previous functional data and structural data to guide definition of patches of interest which were placed in the left and right pre- and postcentral gyri as well as in the left and right superior frontal gyrus and the inferior frontal sulcus. The average cortical thickness was determined for each region of interest (ROI) and participant and results were subjected to statistical analysis using between group t-tests or Pearson correlation.

## Results

### Whole cortex analysis

**Figure 1:**
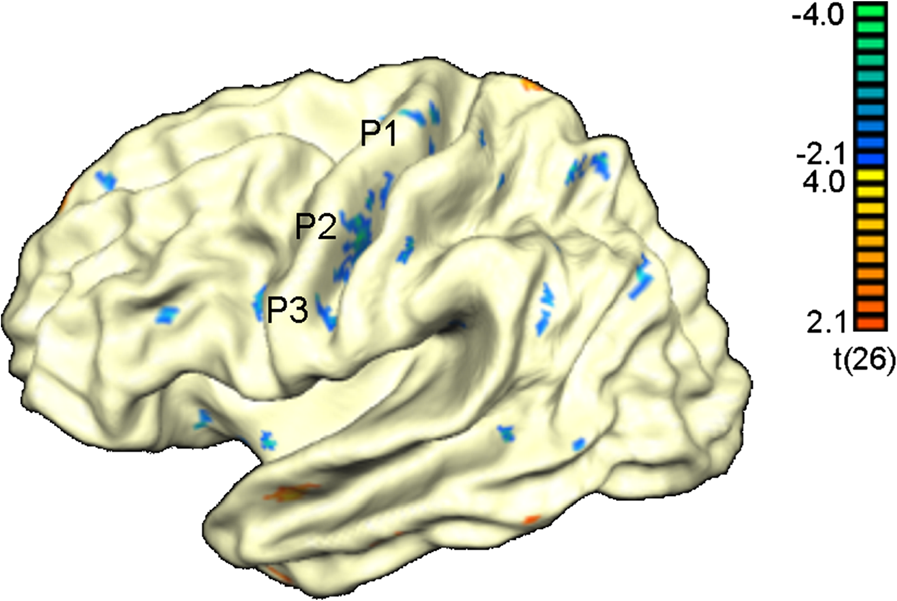
**Results from the whole cortex analysis.** Areas with significant thinning in ALS are depicted blue whereas areas with increased cortical thickness are shown in red. **P1**, **P2**, **P3** refer to patches of interest described further in Figure 2.

Results at the vertex level are shown in Figure 1 with blue colors indicating regions with significantly reduced cortical thickness and red regions indicating regions in which cortical thickness was increased with regard to the normal participants.

Table 2 illustrates the cortical regions that showed significantly reduced cortical thickness in ALS. These included regions in the pre- and postcentral gyrus bilaterally, the SPL bilaterally, the insula bilaterally, the cingulate gyrus and sulcus bilaterally and the angular/supramarginal gyri and regions in the temporal and occipitotemporal cortex. Thus, cortical thinning extended well beyond the sensorimotor cortex.

**Table 2:** Regions of reduced cortical thickness in ALS compared to control participants

**Location**	**# vertices**	**Central vertex**	**BV X**	**BV Y**	**BV Z**	**BA**
**LH precentral G**	51	30255	156.2	70.2	155.4	**4**
**LH precentral G**	132	7509	142.8	91.4	169.1	**4**
**LH precentral G**	24	7961	137.1	104.4	177.4	**6**
**LH precentral S**	23	30468	124.1	104.7	167.6	**6**
**LH postcentral G**	43	7932	141.8	98.5	176.7	**1**
**LH postcentral G**	17	27991	165.4	74.4	159.1	**7**
**LH postcentral G**	12	27754	169.4	82.3	159.5	**7**
**LH postcentral G**	21	8232	151.0	92.6	176.2	**40**
**LH SPL**	39	28075	186.2	80.2	152.2	**7**
**LH IPL**	17	19218	196.2	99.7	162.6	**39**
**LH angular G**	17	34659	178.3	92.8	169.3	**39**
**LH angular G**	27	33840	176.7	102.4	176.5	**39**
**LH insula**	12	12243	110.9	131.2	153.9	**na**
**LH insula**	12	779	119.3	134.5	158.6	**na**
**LH insula**	20	12358	124.7	133.1	162.5	**na**
**LH supramarginal G**	16	33153	160.9	106.5	172.6	**40**
**LH uncus**	13	38776	144.5	139.2	145.6	**na**
**LH cingulate S**	11	37338	98.8	128.6	133.4	**11**
**LH cingulate S**	14	40060	129.0	88.1	137.6	**24**
**LH cingulate G**	13	37611	96.7	116.0	134.5	**24**
**LH cingulate G**	17	25234	168.8	97.1	134.1	**23**
**LH calcarine S**	12	22417	186.8	127.0	151.0	**17**
**RH precentral G**	23	6950	166.0	64.9	122.5	**4**
**RH precentral S**	17	29332	136.2	80.8	100.8	**4**
**RH postcentral G**	19	1638	160.1	85.3	86.0	**7**
**RH postcentral G**	56	24668	152.6	94.7	78.2	**40**
**RH inf. frontal S**	15	37703	94.7	115.1	89.2	**44**
**RH SPL**	15	1788	183.9	76.5	103.2	**7**
**RH lat. occipitotemp. G**	16	21928	198.1	140.5	88.1	**37**
**RH inf. temporal G**	15	22236	196.8	132.1	83.1	**20**
**RH sup. temporal S**	14	21134	149.6	135.8	79.0	**21**
**RH insula**	31	5792	155.4	109.9	94.8	**na**
**RH lateral S**	19	6021	160.5	120.7	80.3	**42**
**RH lateral S**	11	25291	170.9	105.4	80.5	**40**
**RH lateral S**	14	23138	164.4	113.9	91.1	**42**
**RH lateral S**	67	39268	138.9	132.5	91.0	**na**
**RH sup. frontal G**	48	2234	95.3	99.8	120.4	**8/9/10**
**RH cingulate G**	11	11154	175.3	114.5	122.5	**30**
**RH precuneus**	21	17430	182.7	120.3	120.9	**31**
**RH calcarine S**	17	17781	185.8	125.9	104.8	**17**
**RH med. occipitotemp. G**	15	1082	197.2	131.8	118.9	**19**
**RH med. occipitotemp. G**	24	17728	178.6	135.0	105.4	**19**
**RH lat. occipitotemp. G**	45	16368	168.8	145.6	95.5	**20/37**

LH, RH: left, right hemisphere; size of the region is given as number of vertices, regions with 10 or less vertices are left out; the number of the central vertex and its x,y,z coordinates as provided by Brain Voyager are also given; BA: Brodmann area; na: not available; G: gyrus; S: sulcus.

There were few regions showing increased thickness in ALS (see Table 3) including parts of temporal cortex and cingulate cortex.

**Table 3:** Regions of increased cortical thickness in ALS compared to control participants

**Location**	**# vertices**	**Central vertex**	**BV X**	**BV Y**	**BV Z**	**BA**
**LH sup. temporal G**	34	2693	129.7	137.9	177.8	**38**
**LH med. temporal G**	14	16528	147.6	143.4	186.0	**20**
**LH inf. temporal G**	22	14419	126.9	162.3	164.5	**38**
**LH sup. frontal G**	11	37020	81.9	89.2	135.0	**9**
**LH SPL**	19	27475	176.0	66.1	139.2	**5**
**LH cingulate G**	14	39937	118.3	98.4	133.0	**24**
**LH cingulate G**	23	25129	158.7	102.3	132.9	**23**
**LH precuneus**	17	21857	177.7	123.3	137.1	**17**
**LH cingulate G**	31	22280	167.9	129.8	140.3	**30**
**LH hippocampus**	39	13505	130.4	136.9	150.0	**na**
**RH insula**	12	38050	119.0	132.1	97.3	**na**
**RH orbital G**	20	34931	106.5	37.6	101.2	**11**
**RH cingulate G**	79	17828	170.9	128.0	116.6	**30**

LH, RH: left, right hemisphere; size of the region is given as number of vertices, regions with 10 or less vertices are left out; the number of the central vertex and its x,y,z coordinates as provided by Brain Voyager are also given; BA: Brodmann area; na: not available; G: gyrus; S: sulcus.

### Region of interest analysis

Both, left and right precentral cortex showed a highly significant reduction of the cortical thickness in ALS (left: t(26) = 3.76, p < 0.001; right: t(26) = 3.81, p < 0.001). The left and right postcentral gyrus again showed a highly significant reduction of the cortical thickness in ALS (left: t(26) = 3.24, p < 0.005; right: t(26) = 3.51, p < 0.003). The superior frontal gyrus showed significant thinning in ALS on the left (t(26) = 3.2, p < 0.005) and right (t(26) = 3.8, p < 0.001). The inferior frontal sulcus also showed significant thinning on the left (t(26) = 3.29, p < 0.003) and right (t(26) = 2.36, p < 0.03).

**Figure 2:**
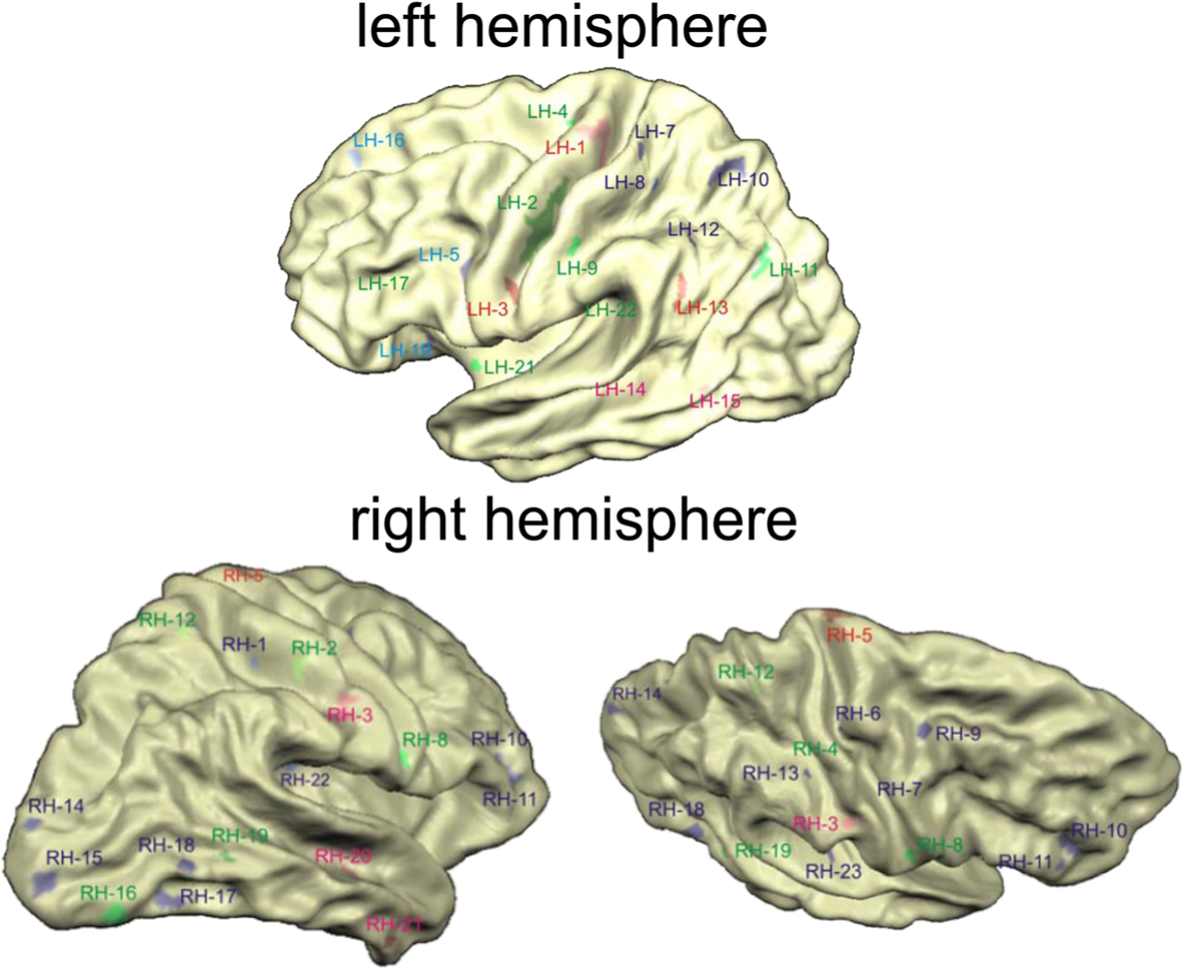
**Views of the left (upper part) and right (lower part) hemispheres with labels for the patches that showed significantly reduced cortical thickness in ALS patients.** See Figure 3 for results for selected patches.

**Figure 3:**
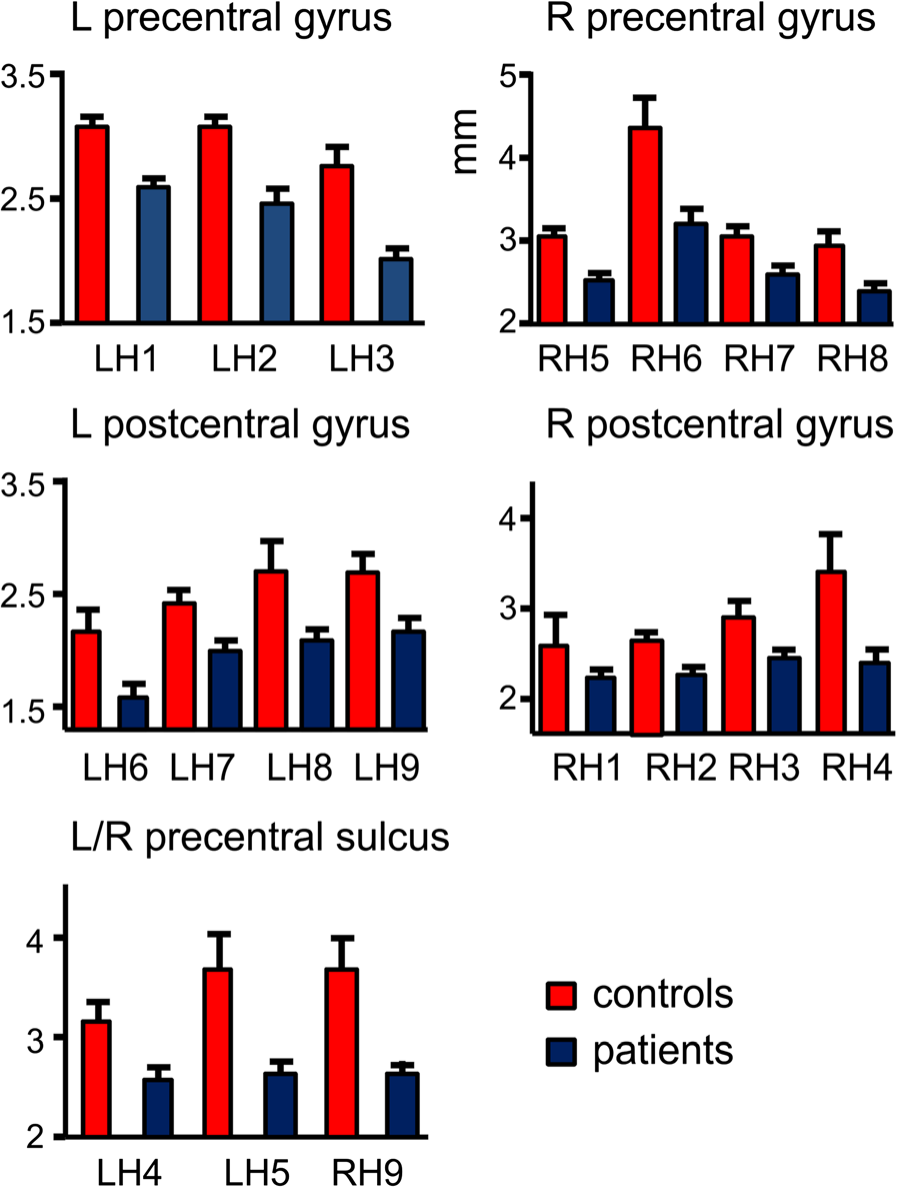
**Illustration of reduction of cortical thickness for several patches in the pre- and postcentral gyri and the precentral sulcus of both hemispheres.** In each case, a reduction of cortical thickness in ALS becomes apparent.

To further illustrate the reduction of cortical thickness in the pre- and postcentral gyri and the precentral sulci, we computed cortical thickness for a number of patches (see Figure 2 for location and Figure 3 for numerical results). Interestingly, LH3 which was located in the area of the bulbar representations numerically showed a more pronounced but non-significant reduction in patients with bulbar involvement (1.7 mm compared to 2.1 mm in non-bulbar patients).

We repeated these ROI analyses with age, sex, and disease duration as covariate using the procedure UNIANOVA of SPSS instead of a *t*-test. This did not change the pattern of results.

### Correlations with clinical data

**Figure 4:**
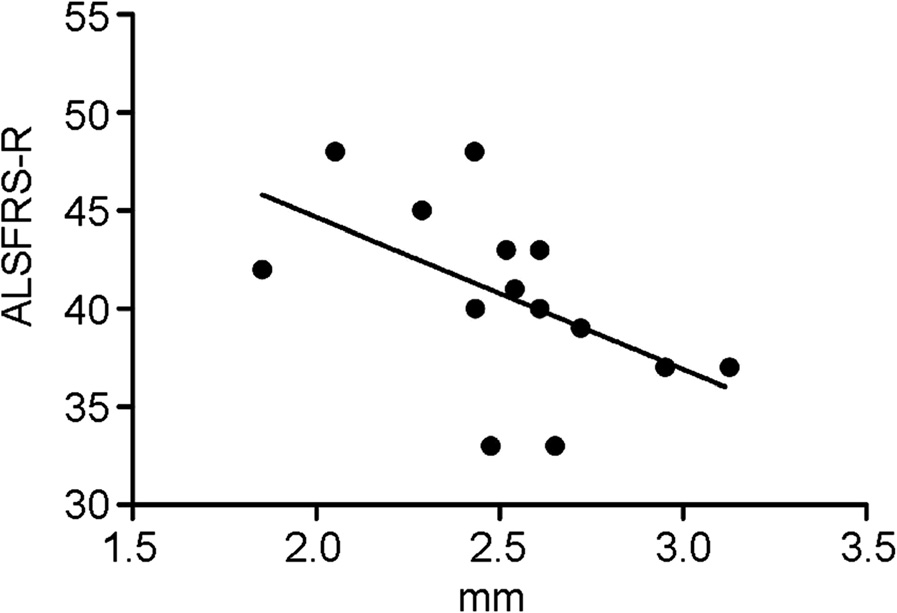
**Relation of clinical severity and cortical thickness of the right frontal gyrus.** There was a negative correlation between ALSFRS-R score and cortical thickness.

Cortical thickness of the ROIs showed a correlation with ALSFRS-R in the right superior frontal gyrus (r = -0.534, P < 0.05, Figure 4) and the right inferior frontal gyrus (r = -0.535; p < 0.05). Cortical thickness of the right inferior frontal sulcus correlated with progression score (r = 0.591; p < 0.03).

We did not find any systematic relationship between cortical thickness and the scores assessing spasticity and reflexes.

Finally, we also did not observe a correlation between age and cortical thickness in any region.

## Discussion

Expectedly, the current study revealed cortical thinning of the primary motor cortex bilaterally in ALS. Importantly, atrophy was also found for the postcentral gyrus bilaterally demonstrating that ALS extends beyond the motor system. These results resonate well with previous findings using voxel-based morphometry [11-13,40-42]. The current analysis of cortical thickness also pointed to an involvement of additional extramotor areas including, for example, the cingulate cortex, as well as regions in the temporal and occipital cortex. That the CTA findings of the current study are not spurious is suggested by other recent studies [28-30] as well as by reports of functional changes implicating the same areas. For example, marked alterations of early visual event-related potential components [43] show that visual cortical areas are indeed altered in the ALS disease process. Also, impaired initiation and inhibition of movements implies the supplementary motor area and right prefrontal cortex [8,44,45].

These findings could guide further neuropathological investigations in ALS in order to pinpoint the underlying histological changes. A marked loss of large pyramidal cells and Betz cells in conjunction with astrocytic gliosis has been described in motor cortex in ALS [25,46]. Other neuropathological studies have shown loss of parvalbumin-positive interneurons in ALS irrespective of the degree of loss of Betz-cells in the motor cortex [47]. Loss of GABAergic interneurons in the primary motor cortex and beyond (dorsolateral prefrontal cortex and anterior cingulate cortex) has also been documented [48]. According to the present data extramotor involvement in ALS is widespread also involving brain areas in the frontal, parietal, temporal and occipital lobe. It remains to be seen whether neuropathological changes are equivalent in all of these regions. Also, it is important to note that the patients involved in the current study were affected only mildly or moderately. Application of cortical thickness analysis in more advanced disease is therefore warranted.

A previous study using MRI-based cortical thickness analysis in ALS used a region-of-interest approach rather than a whole cortex analysis [27]. In contrast to the present results, this study found a significant thinning of the precentral but not postcentral gyrus in ALS. The thickness of the precentral gyrus was determined to be 2.15 mm in normal controls which compares to about 2.8 mm found in neuropathological studies [26]. The current data more closely resemble numbers usually found in post-mortem studies. Another recent study investigated primary lateral sclerosis and focussed on cortical thickness of the primary motor cortex in the area of the hand-knob [31]. Patients showed thinning of the motor cortex but not of the primary sensory cortex. The present investigation as well as other recent studies [28-30] significantly extends these previous findings by using a whole cortex approach supplemented by regions of interest analysis.

Interestingly, cortical thickness in the right superior frontal gyrus and in the right inferior frontal gyrus showed a negative correlation with the ALSFRS-R score. This is counter-intuitive, as lower ALSFRS-R scores indicate worse clinical status and, thus, one would expect lower scores to be associated with cortical thinning. It has to be kept in mind, however, that the ALSFRS-R score assesses global functionality of the patient with a strong focus on motor functions and that the brain areas which showed correlations in the present study were not primary motor. In fact, we did not observe any correlations of the ALSFRS-R score to cortical thickness in the precentral gyrus. A recent study investigated cortical thickness and used scores for upper motor neuron and lower motor neuron involvement [31]. In this study, a regionally specific thinning (e.g., bulbar part of the motor cortex for bulbar symptoms) was described in correlation with upper motor neuron signs.

A few brain regions showed an increase of cortical thickness in ALS, some of them even adjacent to regions showing thinning. None of the VBM studies published on ALS reported increases of grey matter density and it is unclear whether such changes were assessed. While the whole cortex statistics used in the present study were properly safeguarded against type 1 error by correction for multiple comparisons using cluster-size thresholding, it is not impossible that one or the other brain area with increased cortical thickness represents a “false positive”. However, we do not believe that this can explain the observed pattern. Interestingly, the areas with increased thickness are located mainly in temporal and medial frontal cortex (parts of the cingulate cortex). One possibility is that the observed increases are related to compensatory mechanisms. For example, we have observed an increase of cortical thickness in brain areas related to visuospatial processing and memory in subjects that did not learn to read (unpublished), which we took as indicating a greater reliance of illiterates on visuospatial and memory processes. An interplay between frontal and temporal structures is often seen in functional imaging studies of tasks involving executive and working memory functions [49-52] and changes in anatomical connectivity between frontal and temporal structures have been documented in ALS [15]. Moreover, a pattern of hypo- and hyperactivation in a motor task has been observed recently in relation to cortical atrophy in ALS [53]. If structural loss (cortical thinning) in one part of this system is related to structural gain (cortical thickening) in the connected areas remains to be tested in further studies.

### Cortical thickness vs. voxel-based morphometry

This data set has been analyzed previously using VBM [13] which had revealed widespread differences between ALS and normal controls in areas including the right (but not left) precentral gyrus, the postcentral gyrus bilaterally, the inferior parietal lobule bilaterally, and the medial and superior frontal gyri bilaterally. Both, VBM and CTA, showed a correlation between disease severity as reflected by ALSFRS-R and the integrity of the right frontal cortex. CTA appeared more sensitive than VBM in the detection of involvement of extramotor areas. Also, in contrast to the VBM-study the current analysis revealed the left primary motor cortex and supplementary motor cortex. This suggests that cortical thickness analysis is more sensitive to subtle changes of cortical anatomy than VBM. Bermudez et al. [54] similarly reported on differences between VBM and CTA in a study of anatomical changes in professional musicians and underscored the utility of CTA.

One further advantage of CTA is the fact that it measures a meaningful parameter, i.e. cortical thickness, that readily compares to measurements obtained in neuropathological specimens. The fact that the current measurements (about 2.9 mm for the motor cortex in normal participants) were almost identical to values obtained using neuropathological methodology [26] is reassuring. By contrast, VBM determines grey matter density and it is unclear how this translates into actual anatomical changes [54]. On the other hand, determination of cortical thickness is considerably more time-consuming and requires more user interaction than VBM which can be highly automatized.

### Limitations and prospects

Several limitations of the current study should be mentioned. First, the number of participants per group was relatively small and thus the whole cortex analysis obviously is problematic. A larger data base of normal participants and patients would be desirable. This could be used to test the cortical thickness of individual patients in selected regions of interest such as the pre- and postcentral gyri against the distribution of the group, for example using the test statistic proposed by Crawford and Garthwaite [55]. Such an approach might prove clinically useful in cases in which involvement of the upper motor neuron is not yet apparent. To capture the dynamics of the disease, longitudinal studies are desirable. Recently, tools have been developed to assess changes of cortical thickness over time [56].

## Conclusions

The present study adds to previous investigations in showing that the cortical involvement in ALS extends considerably beyond the motor system. The task for the future is to establish a relation between this involvement and motor, cognitive, and emotional deficits in ALS. Initial steps in this direction have already been taken in a recent publication [57].

## Abbreviations

ALS: amyotrophic lateral sclerosis; ALSFRS-R: amyotrophic lateral sclerosis functional rating scale – revised; CTA: cortical thickness analysis; MRC: medical research council; MRI: magnetic resonance imaging; VBM: voxel-based morphometry.

## Competing interests

The authors declare that the research was conducted in the absence of any commercial or financial relationships that could be construed as a potential competing interest.

## Authors’ contributions

JT: Performed the analyses, wrote first draft of the manuscript. HJ: participated in the analysis and revised the manuscript critically for important intellectual content. TP: recorded the data and revised the manuscript critically for important intellectual content. JG: recruited the patients, performed the clinical staging and revised the manuscript critically for important intellectual content. BM: participated in the analyses and revised the manuscript critically for important intellectual content. RD: participated in patient recruitment, study design and revised the manuscript critically for important intellectual content. TFM: conceived the study and wrote the final version of the manuscript. All authors read and approved the final manuscript.

## Pre-publication history

The pre-publication history for this paper can be accessed here:

http://www.biomedcentral.com/1471-2377/13/148/prepub
